# A tubulin alpha 8 mouse knockout model indicates a likely role in spermatogenesis but not in brain development

**DOI:** 10.1371/journal.pone.0174264

**Published:** 2017-04-07

**Authors:** Christine P. Diggle, Isabel Martinez-Garay, Zoltan Molnar, Martin H. Brinkworth, Ed White, Ewan Fowler, Ruth Hughes, Bruce E. Hayward, Ian M. Carr, Christopher M. Watson, Laura Crinnion, Aruna Asipu, Ben Woodman, P. Louise Coletta, Alexander F. Markham, T. Neil Dear, David T. Bonthron, Michelle Peckham, Ewan E. Morrison, Eamonn Sheridan

**Affiliations:** 1 School of Medicine, St James's University Hospital, University of Leeds, Leeds, United Kingdom; 2 Department of Physiology, Anatomy and Genetics, University of Oxford, Oxford, United Kingdom; 3 School of Medical Sciences, University of Bradford, Bradford, United Kingdom; 4 School of Biomedical Sciences, University of Leeds, Leeds, United Kingdom; 5 School of Molecular and Cellular Biology, University of Leeds, Leeds, United Kingdom; 6 Yorkshire Regional Genetics Service, St. James's University Hospital, Leeds, United Kingdom; Nanjing Medical University, CHINA

## Abstract

Tubulin alpha 8 (Tuba8) is the most divergent member of the highly conserved alpha tubulin family, and uniquely lacks two key post-translational modification sites. It is abundantly expressed in testis and muscle, with lower levels in the brain. We previously identified homozygous hypomorphic *TUBA8* mutations in human subjects with a polymicrogyria (PMG) syndrome, suggesting its involvement in development of the cerebral cortex. We have now generated and characterized a *Tuba8* knockout mouse model. Homozygous mice were confirmed to lack Tuba8 protein in the testis, but did not display PMG and appeared to be neurologically normal. In response to this finding, we re-analyzed the human PMG subjects using whole exome sequencing. This resulted in identification of an additional homozygous loss-of-function mutation in SNAP29, suggesting that SNAP29 deficiency, rather than TUBA8 deficiency, may underlie most or all of the neurodevelopmental anomalies in these subjects. Nonetheless, in the mouse brain, Tuba8 specifically localised to the cerebellar Purkinje cells, suggesting that the human mutations may affect or modify motor control. In the testis, Tuba8 localisation was cell-type specific. It was restricted to spermiogenesis with a strong acrosomal localization that was gradually replaced by cytoplasmic distribution and was absent from spermatozoa. Although the knockout mice were fertile, the localisation pattern indicated that Tuba8 may have a role in spermatid development during spermatogenesis, rather than as a component of the mature microtubule-rich flagellum itself.

## Introduction

Tubulin alpha 8 (TUBA8) belongs to the alpha tubulin gene family, of which there are eight members in man and seven in mouse [1]. Alpha and beta tubulins form heterodimers to generate protofilaments that make up microtubules. These hollow 25-nm diameter tubes are key components of the cytoskeleton and are dynamic structures that are involved in many cell processes. During mitosis they are a main component of the spindle, whereas during interphase they have a structural support role, but can also be involved in intracellular trafficking and cell motility.

Compared to other alpha tubulins, TUBA8 was only described relatively recently [2], and there is still very little known about its function. Although the alpha tubulin protein sequences are all highly conserved, Tuba8 is the most divergent. Indeed the human TUBA8 protein sequence has a higher level of similarity to the mouse protein than to the other human alpha tubulins, and both species share the same gene structure and synteny [1]. Notably, two regions of particular sequence divergence contain key sites for post-translational modification in the other alpha tubulins [2–4]. A ten amino acid peptide sequence containing a site of lysine acetylation (K40) in other alpha tubulins is absent in TUBA8, and this lysine is replaced by alanine. Acetylation of K40 is associated with increased microtubule stability [5–6], and has been proposed to contribute to the modulation of microtubule motor activity [7–8]. TUBA8 does however have a lysine residue two amino acids downstream, which is absent in the other alpha tubulins; whether K42 can also be acetylated with functional consequences is not known. The second region of sequence divergence is at the carboxy-terminus, where TUBA8 terminates with a phenylalanine instead of the usual tyrosine. The removal of the alpha tubulin C-terminal tyrosine to reveal a charged glutamate is again associated with changes to microtubule stability and function [9–12]. Together, these differences suggest a unique role for TUBA8 in microtubule biology.

Tubulins are expressed in every cell type. Some isoforms are expressed ubiquitously whilst others, including TUBA8, have a tissue-restricted distribution. The identification of patients with mutations in alpha and beta tubulin genes has implicated the proteins as having an important role in the developing brain. To date, patients with neurological phenotypes have been found to harbour mutations in three beta tubulin genes (*TUBB2B*, *TUBB3*, *TUBB5*) and two alpha tubulin genes (*TUBA1A*, *TUBA8*) [13–17]. We previously found that patients with developmental delay, polymicrogyria (PMG) and optic nerve hypoplasia had a mutation in the splice site polypyrimidine tract of TUBA8, in two families, possibly of common ancestry [17]. Interestingly this phenotype was inherited in an autosomal recessive manner, whilst previously reported tubulin gene mutations behaved as autosomal dominant traits. No additional patients with *TUBA8* mutations have so far been reported.

Although TUBA8 is an under-studied member of the tubulin family, its mRNA expression profile suggests a role in the testis and muscle, with highest levels in these tissues. There are much lower levels in the brain [2, 18]. One possible explanation for this latter observation is that TUBA8 could be limited to a key subset of cells that control normal brain development [19]. This nevertheless contrasts with TUBA1A, TUBB2B, TUBB3 and TUBB5, whose roles include neuronal migration and axon guidance which impact on final brain structure and function, and where high expression levels are present in the adult or developing brain [18].

Given this gap in understanding of the biology of Tuba8, we sought to investigate further these functional questions through the generation of a mouse knockout model.

## Materials and methods

### Generation of a Tuba8 conditional knockout mouse

**Fig 1:**
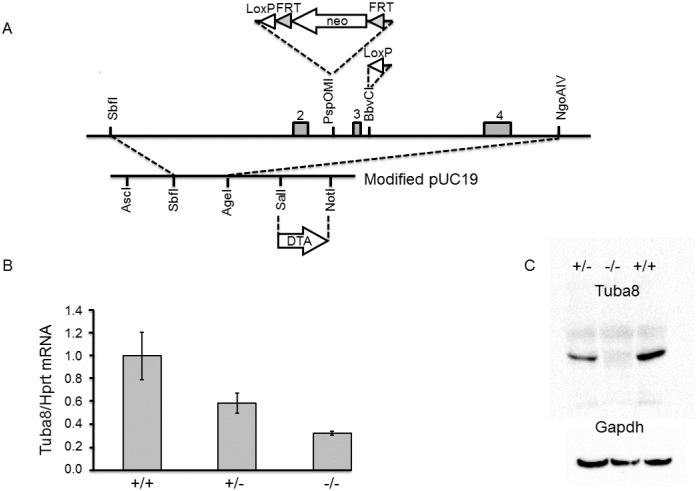
Generation of a *Tuba8* knockout allele. A) Design of the targeting construct. A 9.1-kb SbfI–NgoAIV genomic fragment was directionally cloned into the SbfI+AgeI-restricted pUC19 vector. The shaded numbered boxes depict exons 2–4. The arrows represent the location and direction of expression of the selectable markers. The neomycin (*neo*) cassette, obtained from PGKneobpA [22], was inserted at the PspoMI site. The FRT sites (shaded arrow heads) flanked the neomycin positive selection cassette, whilst one LoxP site flanked one side of the *neo*, and the second was inserted into the BbvCI site in intron 3 (unshaded arrow heads). The diphtheria toxin negative selection cassette, PGK-DTA (kindly provided by P. Soriano, Fred Hutchinson Cancer Research Center, Seattle, WA), was inserted between the SalI and NotI sites of the modified pUC19 backbone. B) Quantitative reverse transcription PCR. Tuba8 mRNA levels in testis were determined from four animals of each Tuba8 genotype, wild type (+/+), heterozygous (+/−) and homozygous (−/−), and normalised to Hprt levels. Bars indicate mean ± 1 s.d. Statistical significance was reached for all combinations of genotypes using an unpaired t-test (p<0.05). C) Protein analysis using western blotting. Protein lysates from testis samples used four animals of each genotype: wild type (+/+), heterozygous (+/−) and homozygous (−/−), in lanes 1–3 respectively, were probed with the Bioserv Tuba8 mouse monoclonal antibody. A band of approximately 55 kDa was detected in the wild type and heterozygous samples. The blots were subsequently probed for Gapdh expression as a loading control to quantify expression.

Tuba8-containing genomic clones were isolated from mouse strain 129S6/SvEvTac by screening the RPCI-21 PAC library (Medical Research Council (MRC) Geneservice). A fragment containing exons 2–4 was cloned into pUC19 (Fig 1a). Positive and negative selection cassettes were added, with FRT sites positioned to allow deletion of the positive selection cassette, and LoxP sites for deletion of *Tuba8* exon 3 (Fig 1a).

The construct was linearised and electroporated into E14.1a mouse embryonic stem (ES) cells (Geneta, University of Leicester, UK). G418-selected ES cells were screened for correctly targeted construct by Southern blotting. ES cell clones were injected into C57BL/6J blastocysts and transferred to pseudo-pregnant mothers (Geneta). The resultant chimeras were mated with a FLP deleter strain maintained on a C57BL/6J background (Geneta).

Removal of *Tuba8* exon 3 to generate the null allele was achieved by breeding with animals with ubiquitous embryonic Cre recombinase expression. In the PGK-Crem mouse line the Cre recombinase expression was driven by the PGK-1 promoter. This line was a kind gift from Dr Ian Rosewell, London Research Institute, CR-UK. Experimental animals were housed in specific pathogen free conditions, free access to diet and water, and maintained with a 12 hour light and dark cycle. Mice were euthanised by cervical dislocation or anaesthetic overdose, and embryos harvested and decapitated. A project licence (40/3470) approving this work was granted by the Home Office (United Kingdom) as required by the Animals (Scientific Procedures) Act 1986.

### Transcript analysis

For quantitative reverse transcription PCR, total RNA was DNaseI-treated (Ambion, Huntingdon, UK) prior to first strand synthesis. PCR was performed using the SYBR Green PCR master mix (ABI, Warrington, UK) and an ABI 7500. Primer sequences are provided in the S1 Appendix.

### Antibodies

Commercial antibodies used were TUBA8, which was raised against the entire protein (SAB5300189, Sigma), Acetylated tubulin clone 6-11B-1 (Sigma), and tyrosinated tubulin clone YL1/2 (AbDSetotec). A custom mouse monoclonal IgG purified was generated against the most divergent region encompassing amino acids 35–45 (TFGTQASKIND) of murine Tuba8, which is located in exon 2 (Bioserv, Sheffield, UK).

### Western blot analysis

Cell and tissue extracts were prepared by homogenizing samples in Laemmli buffer. Protein extracts were separated on SDS-PAGE gels, and blotted onto PVDF membrane. Membranes were blocked in 5% non-fat milk powder in PBS with 0.1% Tween 20 (PBS-T). Antibodies were incubated in 1% non-fat milk in PBS-T with washing in PBS-T. Signal development and detection used the SuperSignal Chemiluminescent substrate (Pierce, Cramlington, UK), and Imagelab software (Biorad).

### Histological analysis

Tissues were fixed overnight in 4% paraformaldehyde in PBS, wax-embedded and 5-μm sections cut. For immunolabelling, sections were dewaxed, rehydrated, subject to antigen retrieval in 10 mM citrate buffer, pH 6.0, and peroxidase blocked using 0.3% hydrogen peroxide, before protein blocking using casein. Detection used the EnVision DAB system (Dako, Ely, UK), unless the primary antibody was derived from mouse, in which case the mouse-on-mouse immunolabelling kit was used (Vector Laboratories, Peterborough, UK). Histological identification of tubule stages and germ cell types was achieved using Russell et al [20] as a guide.

### Statistical testing

Unpaired t-tests were used to determine statistical significance of transcript and protein expression using GraphPad software. Chi-squared tests were used to investigate litter genotype numbers.

### Exome sequencing

Written informed consent was obtained, ethical approval was given by the Leeds (East) Research Ethics Committee (reference 07/H1306/113). Exome analysis was performed as part of clinical care, ethical approval did not include publication of Next Generation Sequencing raw data files. Exome sequencing libraries were prepared using the SureSelect v5 reagents (Agilent Technologies, Wokingham, UK), and sequenced using 100-bp paired end reads on a HiSeq 2000 (Illumina Inc., San Diego, California, USA). Raw data were demultiplexed using CASAVA v.1.8.2 and sequences in the per-sample FASTQ.gz files were aligned to a reference genome (hg19) using BWA v.0.7.13 (http://bio-bwa.sourceforge.net). SAM/BAM file processing (including duplicate read removal) was performed using Picard v.2.1.1 (http://picard.sourceforge.net) prior to indel realignment, base quality score recalibration and variant discovery using the Genome Analysis Toolkit (GATK) v.2.3.-4Lite.

Identified variants were annotated using Alamut Batch standalone v.1.4.4, (database v.2016.03.04) and filtered using the AgileExomeFilter (http://dna.leeds.ac.uk/agile), essentially as described previously [21].

## Results

### Generation of a Tuba8 knockout mouse

A targeting construct was designed to delete exon 3, in order to generate a *Tuba8* knockout (Fig 1a). Correctly recombined embryonic stem cell clones were identified by Southern blotting (S1 Fig, S1 Appendix). Following removal of the neomycin cassette, the floxed exon 3 was deleted, which was confirmed by PCR genotyping and Sanger sequencing (S1 Fig, S1 Appendix). Since the original *TUBA8*-mutated patients had some residual full-length transcript [17], the possibility of embryonic lethality due to the putatively null mutation in the knockout mouse was examined. Litters produced from heterozygous matings were of normal size, and the ratios of the genotyped offspring did not deviate statistically from those expected (n = 72, chi-squared p = 0.72).

RNA analysis confirmed the deletion of exon 3 and Sanger sequencing showed exon two and four to be spliced consecutively in the knockout (S2 Fig). This resulted in nonsense-mediated decay (NMD) of the transcript, with statistically significant differences in levels between the genotypes (Fig 1b, S2 Fig, S1 Appendix). Tuba8 protein was also absent from the testes of knockout mice, and protein levels were again statistically significantly different between the genotypes (Fig 1c, S3 Fig, S1 Appendix). There was no indication that an aberrant truncated protein was produced from the deleted allele (S3 Fig). Loss of Tuba8 protein was also demonstrated in the lower-expressing brain tissue (S3 Fig, S1 Appendix).

The Tuba8 knockout pups appeared normal and progressed to adulthood. This was unexpected, since the original human patients were severely developmentally impaired, even with their leaky homozygous mutation. Indeed, of the four affected patients only one is currently alive. As Tuba8 is highly expressed in the testis, knockout males and females were mated to determine if the absence of Tuba8 could impair fertility. Animals were able to become pregnant and carried pups to full term delivery, indicating no gross fertility effect on the three litters analysed.

### Histological analysis of the mouse model brain

**Fig 2:**
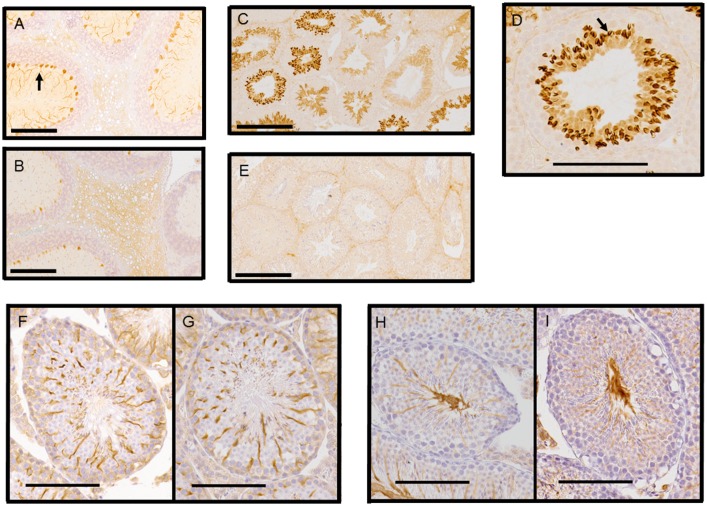
Immunohistochemical analysis of Tuba8 knockout. **A,B.** Cerebellum, stained using the Tuba8 monoclonal (Bioserv). (A, control animal; B, knockout animal) Scale bar = 200 μm. Arrow in part A indicates stronger labelling of dendrites in control tissue. **C-E.** Testis stained using the Tuba8 monoclonal (Bioserv). (C, D, wild-type control, low and higher magnification respectively: C, scale bar = 200 μm; D, scale bar = 100 μm. E, knockout at low magnification. Arrow indicates strong Tuba8 presence peripheral to the nucleus. **F,G.** Staining for tyrosinated alpha tubulin. Control (F) and knockout (G) testis. Scale bar = 100 μm. **H,I.** Staining for acetylated alpha tubulin. Control (H) and knockout (I) testis. Scale bar = 100 μm

A low level of Tuba8 expression in the brain, especially in the cerebral cortex relative to the cerebellum, has previously been described at the RNA level [18]. Immunohistochemical analysis of Tuba8 expression in adult brains was unable to detect any differences in the cortex between the genotypes. However, we were able to demonstrate differences in the cerebellum (Fig 2a and 2b). Here, Tuba8 labelling was found in Purkinje cell dendrites in both the control and knockout; however, there did appear to be a difference in labelling intensity. This was consistent and reproducible across all six control and six knockout brains examined. This suggested that despite some background non-specific immunolabelling, Tuba8 was expressed at detectable levels in Purkinje cells, consistent with the observations of Braun et al. [18].

As we were unable to clearly detect any Tuba8 labelling differences in the cerebral cortex, we sought to examine histological markers of the cortical layers themselves. Mouse models with mutations in other tubulin proteins have all shown aberrations in the cortex [13, 15–16]. It has been suggested that tubulins play an important role in the migration and positioning of neurons during development, which is the cause of the subsequent substantial neurological problems in the patients. Consequently, postnatal (P10) mouse brains were initially investigated for differences in cortical layering between control and knockout, but no consistent alterations were identified. We therefore investigated developing brains at E18.5, to determine whether there was any delay in the migration rate as well as final positioning of neurons. No consistent differences in the embryonic cortex was detected between the control and knockout samples (S4 Fig, S1 Appendix)

### Exome analysis

Due to the absence of histological or immunohistochemical alterations in the cortex in the *Tuba8* knockout animals, we performed further genetic analysis of two of the original *TUBA8*-mutated patients [17], using exome sequencing (summary metrics from these data are reported in S1 and S2 Tables). Standard filtering criteria confirmed the presence of the homozygous *TUBA8* mutation, but also identified, within the autozygous candidate interval (chr22:17841068–25248310), a previously reported homozygous frameshift in *SNAP29* (NM_004782.3 c.487dupA, p.Ser163Lysfs*6) [23]. Bi-allelic mutations in this gene are known to cause CEDNIK syndrome (cerebral dysgenesis, neuropathy, ichthyosis and keratoderma; OMIM: 609528), and these patients have PMG [23–25]. This new data indicated that the PMG in our patients was likely to be due, in whole or in part, to the *SNAP29* rather than the *TUBA8* mutation.

### Tuba8 tissue localisation

Although Tuba8 may not have a dominant role in the developing brain, it is highly expressed in both testis and skeletal and cardiac muscle tissue, and so further investigations were undertaken in these organs.

Tuba8 expression in normal mouse hearts was found to be punctate in the muscle cells and absent from the endothelium (S5 Fig, S1 Appendix). In isolated skeletal muscle fibres Tuba8 was found throughout the microtubule network, and there was a suggested change in nuclei shape in the knockout fibres (S5 Fig, S1 Appendix).

The localisation of testicular Tuba8 was very clearly cell-type restricted (Fig 2c–2e). Specific labelling appeared first mainly in the cytoplasm of step 8 round spermatids in stage VIII. Strong expression was then seen in the step 8 spermatids after spermiation and in stages IX—XII in step 9–12 spermatids. In these cells, staining became particularly intense in the acrosomal region, while the staining for Tuba8 in the cytoplasm diminished a little in stages XI and XII. Thereafter, in the early stages (I–V), expression was lost from the acrosome and nucleus but was maintained in the cytoplasm. Cytoplasmic staining continued into the mid-stages until formation of the cytoplasmic droplet when expression diminished, becoming only faint or absent in stage VII in step 16 spermatids. Thus, following shedding of the cytoplasmic droplet at spermiation in stage VIII, Tuba8 was not present from the immature spermatozoa shed into the lumen. Some non-specific staining was present in the interstitial cells and faintly in some Sertoli cell nuclei, as judged by comparison with the knockout tissue.

The absence of Tuba8 did not noticeably alter the localisation and expression level of total acetylated or tyrosinated tubulins, which were distinct and showed little overlap with Tuba8 (Fig 2f–2i). Tyrosinated tubulin was present at a high level in the Sertoli cell cytoplasm in both control and knockout tissues, and in the flagella of maturation-phase spermatids immediately prior to spermiation (Fig 2f and 2g). Acetylated tubulin showed the same localisation to the flagella of immature spermatozoa and Sertoli cell cytoplasm (Fig 2h and 2i).

Tuba8 was absent from the immature sperm shed into the lumen of the tubules; this contrasts to a recent report in which TUBA8 was described in the flagella of human spermatozoa, and was reduced in asthenozoospermia, at the RNA and protein level [26]. Immunocytochemistry on sperm from the mouse model did label the flagella, but failed to detect a difference between control and knockout mice, suggesting this staining was not Tuba8-specific (S6 Fig, S1 Appendix).

### Functional analysis

In order to detect any functional consequences due to lack of Tuba8, standard behavioural phenotyping tests were performed: open field, modified SHIRPA, grip strength, motor function wheel running, gait analysis, spontaneous alternation, acoustic startle/PPI, and fear conditioning (S1 Appendix). Although some differences were detected in the motor co-ordination of males, this was restricted to the rear left leg and was absent from the rear right leg (S7 Fig). Also the difference was found between the wild type and heterozygous animals, and not between the wild type and homozygous animals, indicating lack of biological relevance. Overall the cognitive and motor function tests did not detect any significant differences between the genotypes in both male and female mice (S1 and S2 Data, S7 Fig).

Since heart tissue contains high levels of Tuba8, primary cardiomyocytes were isolated and used for functional analysis. The myocyte length, width and resting sarcomere length did not significantly differ between the genotypes (S8 Fig, S1 Appendix). Increasing electrical stimulation frequency decreased the time taken for contraction to develop and relax, but Tuba8 status had no significant effects on contraction (S8 Fig, S1 Appendix).

## Discussion

A *Tuba8* knockout mouse model has been generated and validated at the DNA, RNA and protein level. A clear functional consequence of the resulting deficiency of Tuba8 was not however demonstrated. Studies of the normal and knockout mouse did suggest a specialised function for Tuba8 in the testis, judged from its restricted expression pattern. The localisation of Tuba8 did not resemble the patterns seen for other tubulins, [27–33]; instead, it localised to specific cell types at particular developmental time-points. This suggests tight regulatory control, with the factors involved yet to be determined. The juxta-acrosomal localisation during particular stages of spermatogenesis but absence from the acrosome in spermatozoa suggested the possibility that Tuba8 might be important in the biogenesis of this organelle. However, the Tuba8 knockout mice clearly still had sufficient acrosomal function, since homozygous males were fertile. It remains possible that removal of Tuba8 had a more subtle effect on fertility. Evidence from other tubulin studies showed that even with total absence of acetylated tubulin in the testis, animals were still fertile, though some reduction in sperm function was noted when specifically investigated [34].

The predominant Sertoli cell labelling has been documented previously for tyrosinated tubulin, and has been found using pan-alpha, and pan-beta antibodies, as well as those identifying specific beta isoforms TUBB3, TUBB2A, TUBB2C, TUBB5 [27–33]. Sertoli cells have a role in nurturing and structurally supporting the cells undergoing spermatogenesis. Tuba8 appears not to be involved in this support process, and is unlikely to be the predominant dimerization partner of these specific beta variants. Also the general lack of co-localisation of Tuba8 with tyrosinated tubulin is consistent with the unique C-terminal sequence of Tuba8, and suggests it does not undergo tyrosination *in vivo*.

Localisation of acetylated tubulin to spermatozoal flagella has been described previously [30, 34], while the lack of Tuba8 shown here implies that it is not important for generating the microtubules comprising this motile structure. Indeed the lack of Tuba8 had little observable effect on the expression pattern of either tyrosinated or acetylated tubulin in the testis. The increased content of acetylated tubulin in long-lived microtubules is well established, so that one might speculate that microtubules containing high levels of Tuba8 are associated with more dynamic structures. However, the relationship between acetylation status and stability is complex, as acetylation itself does not confer stability, though what its function is has yet to be determined [6, 35]. Furthermore, de-tyrosinated tubulin is associated with microtubule stability, but again is not itself the cause [36]. Consequently, any effect of Tuba8 on microtubule stability remains speculative.

Nevertheless, our observations are consistent with a role for Tuba8 in dynamic structures produced during spermatogenesis. Its presence and subsequent loss from the developing acrosome suggests a role for Tuba8 in guiding the early formation of this organelle. Similarly, the cytoplasmic presence of Tuba8 in elongating spermatids coincides with the elongation of the flagellum and the redistribution of the cytoplasm along the flagellum and away from the nucleus. One possibility therefore might be that testicular Tuba8 forms, or is part of, a microtubular scaffold to allow these dynamic changes in spermatid structure. These changes are absolutely critical to the production of spermatozoa so it can be further speculated that other tubulins perhaps can perform the function of Tuba8 if it is missing, since the knockout mice appeared to have relatively normal spermatogenesis and fertility.

The lack of a mouse KO phenotype resembling that of the patients described in Abdollahi et al [17] prompted us to re-evaluate these subjects using whole exome sequencing. Within the previously defined autozygous region predicted to contain the causative mutation, in addition to the described *TUBA8* loss of function mutation, we identified a mutation in *SNAP29*, previously identified as the cause of CEDNIK syndrome [24]. All the original CEDNIK cases showed a very characteristic pattern of microcephaly and facial dysmorphism with elongated faces, upslanting palpebral fissures, slight hypertelorism, and flat broad nasal root [24]. Palmoplantar keratoderma and ichthyosis were invariant features in these and in a subsequent report [23]. Nerve conduction studies in the original CEDNIK cases revealed a peripheral neuropathy, while retinal electrophysiology demonstrated a macular dysfunction. The three boys we reported had none of the dysmorphic features, the palmoplantar keratoderma, nor the peripheral neuropathy. They had optic nerve hypoplasia rather than macular abnormalities [17]. In retrospect, the eye abnormalities and PMG seen in our cases are reminiscent of the original [24] and subsequent [23, 25] CEDNIK patients. However the absence of the dermatological and other features led us to believe we were dealing with a distinct entity. We now suspect that the main clinical features in our patients were caused by the *SNAP29* mutation rather than the *TUBA8* mutation. It is possible that as CEDNIK is a very rare condition with only a few patients with SNAP29 mutations reported in the literature, the dermatological features may be a variable feature of the disease. Consequently our results indicate that patients without dermatological features should still be considered for potential SNAP29 involvement and diagnosis of CEDNIK. It remains possible that these patients’ TUBA8 deficiency had a modifying effect on the *SNAP29* mutation; however, in the mouse *Tuba8* knockout, no obvious behavioural abnormality was noted.

In summary, the biological function(s) of TUBA8 remain elusive. Our previous suggestion of a role in brain development appears likely to be incorrect. The functional role of Tuba8 in cells undergoing spermatogenesis may be to do with the dynamic changes occurring during spermiogenesis, but this remains to be confirmed.

## Supporting information

S1 Fig Targeting of the Tubulin alpha 8 (Tuba8) gene. (PDF)

S2 Fig Loss of Tuba8 RNA expression in knockout mice. (PDF)

S3 Fig Loss of Tuba8 protein expression in knockout mice. (PDF)

S4 Fig Normal cortical lamination in Tuba8 deficient brains. (JPG)

S5 Fig Tuba8 localisation in muscle. (PDF)

S6 Fig Tuba8 in mouse sperm. (PDF)

S7 Fig Behavioural phenotyping. (PDF)

S8 Fig Isolated cardiomyocyte analysis. (PDF)

S1 Table Summary metrics for exome sequencing assay. (PDF)

S2 Table Variant count for each patient following sequential filtering criteria. (PDF)

S1 Data Mouse phenotyping data 1. (XLSX)

S2 Data Mouse phenotyping data 2. (XLSX)

S1 Appendix Supporting information. (DOCX)
