# Supplementary figures and images for: A tubulin alpha 8 mouse knockout model indicates a likely role in spermatogenesis but not in brain development
Source: PLoS One. 2017 Apr 7;12(4):e0174264. doi: 10.1371/journal.pone.0174264 (PMC5384676; doi:10.1371/journal.pone.0174264)

A

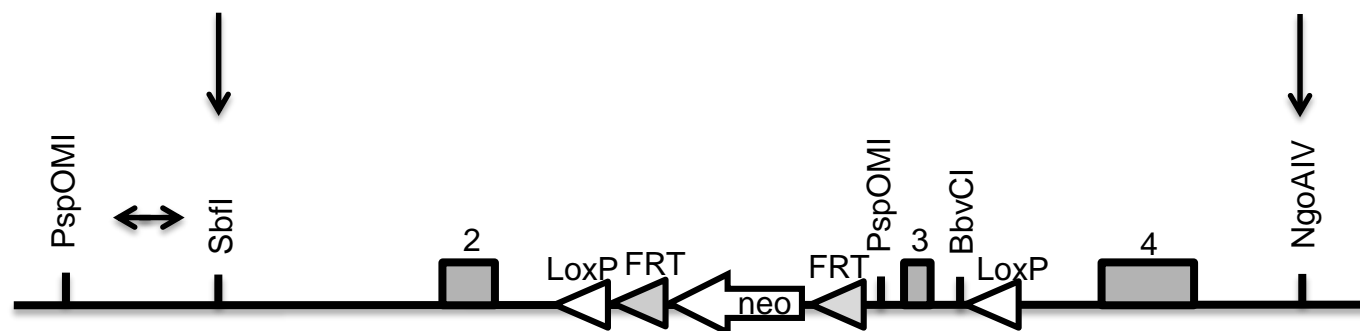

B

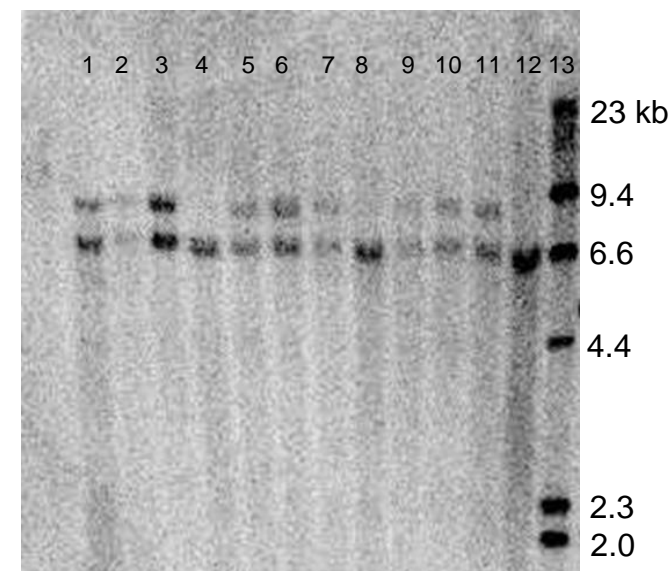

C

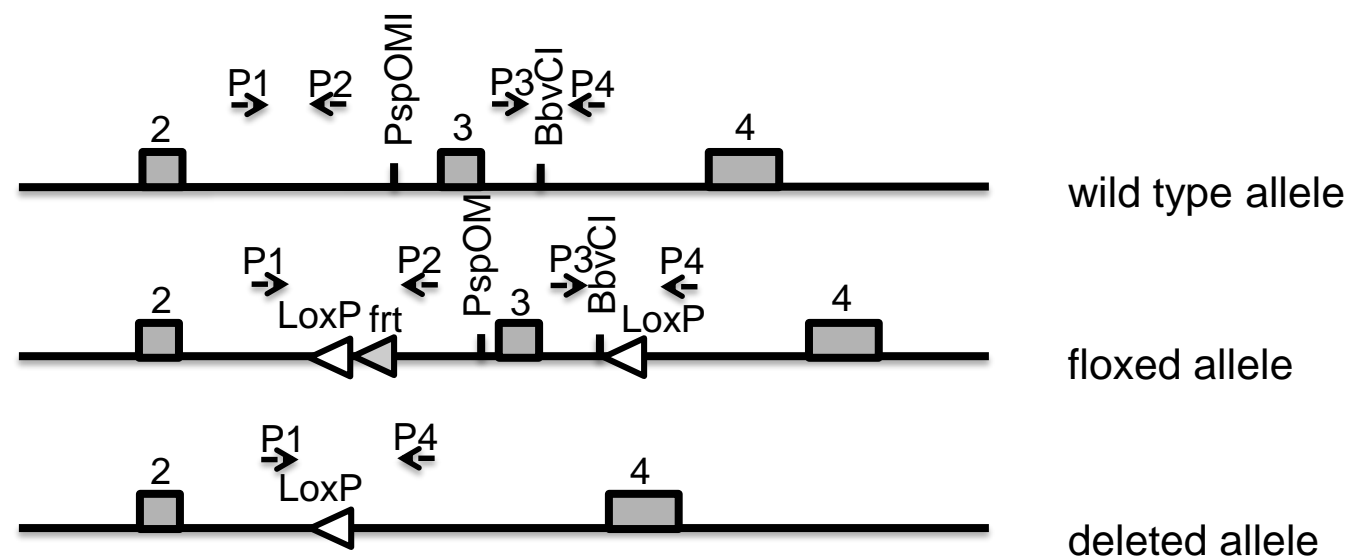

D

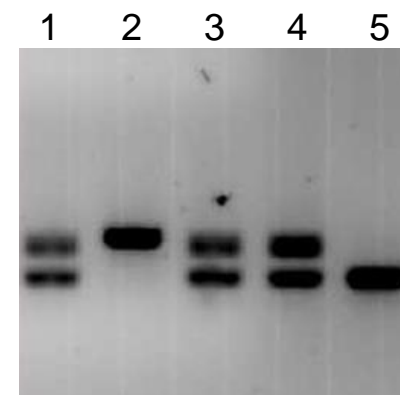

Supplement: S1 Fig — (PDF) [file pone.0174264.s001.pdf]

A

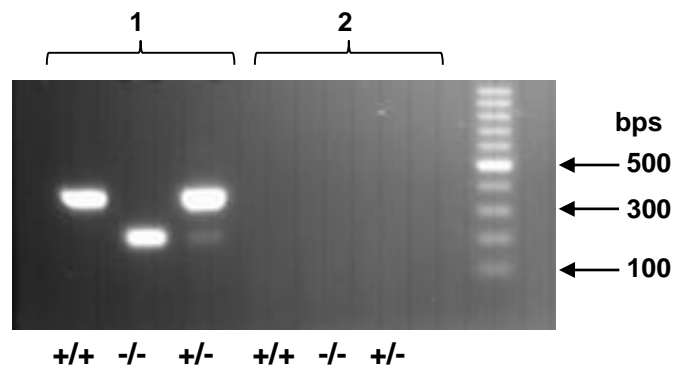

C

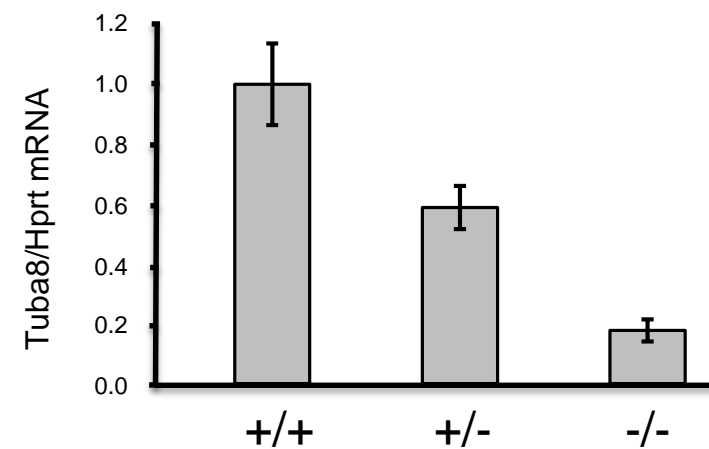

B

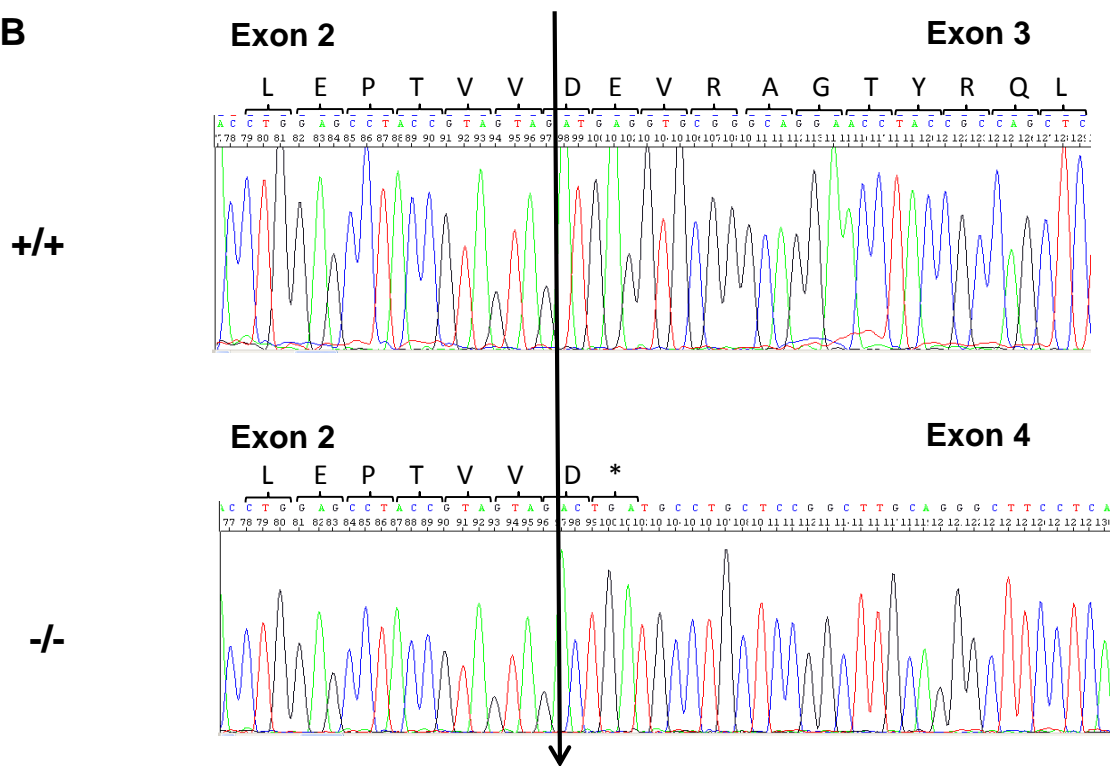

Supplement: S2 Fig — (PDF) [file pone.0174264.s002.pdf]

A

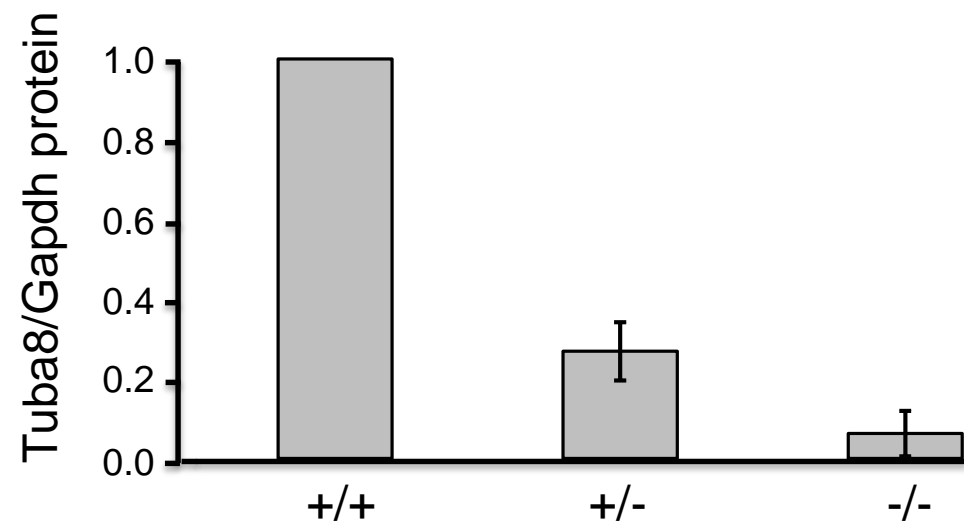

B

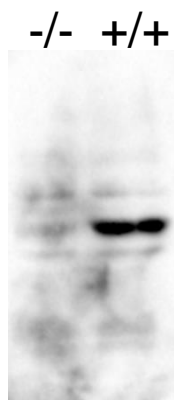

C

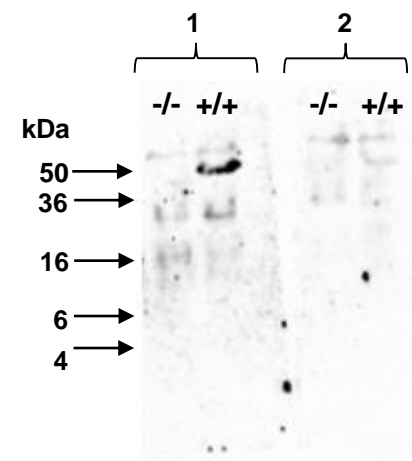

Supplement: S3 Fig — (PDF) [file pone.0174264.s003.pdf]

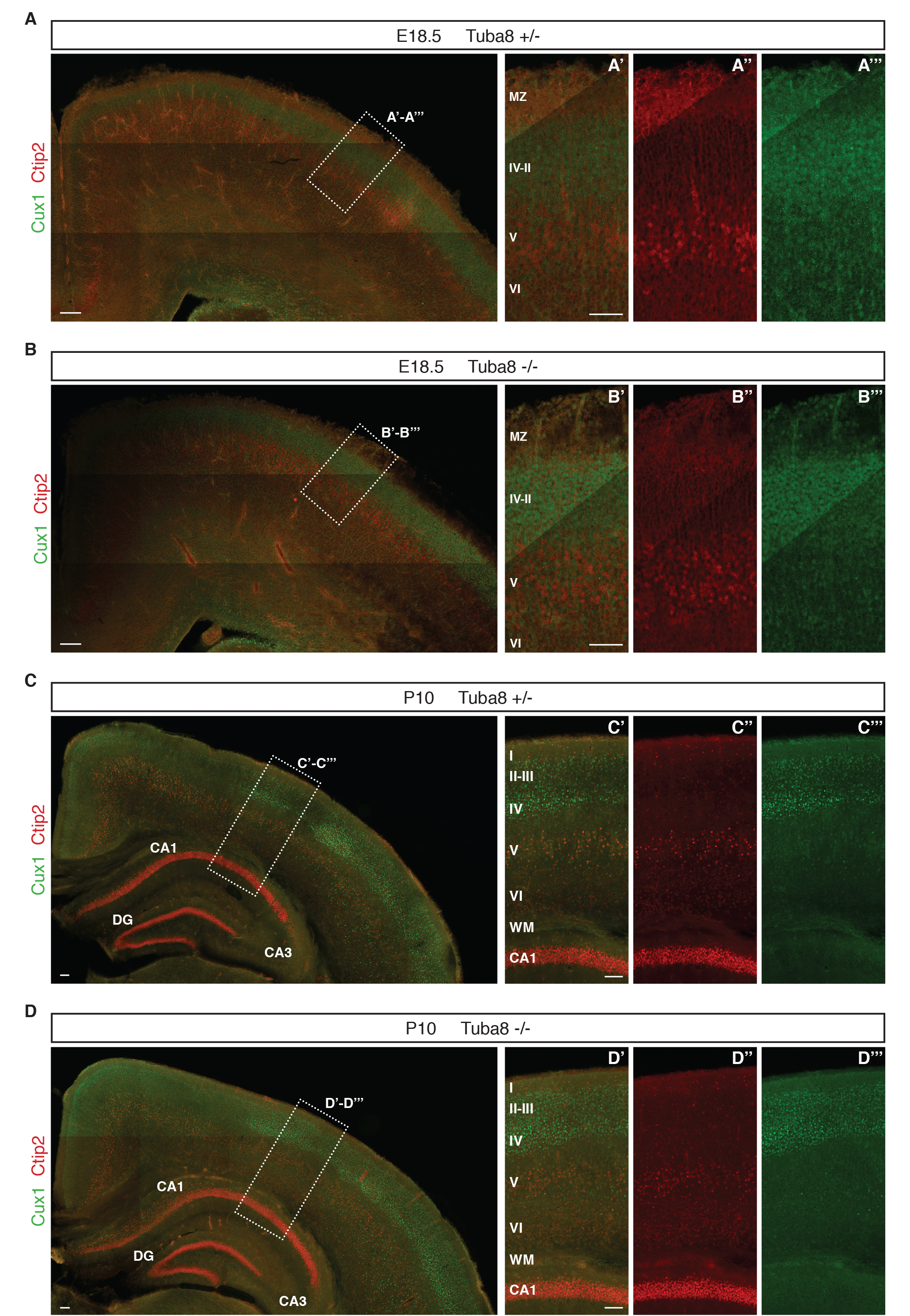

Supplement: S4 Fig — (JPG) [file pone.0174264.s004.jpg]

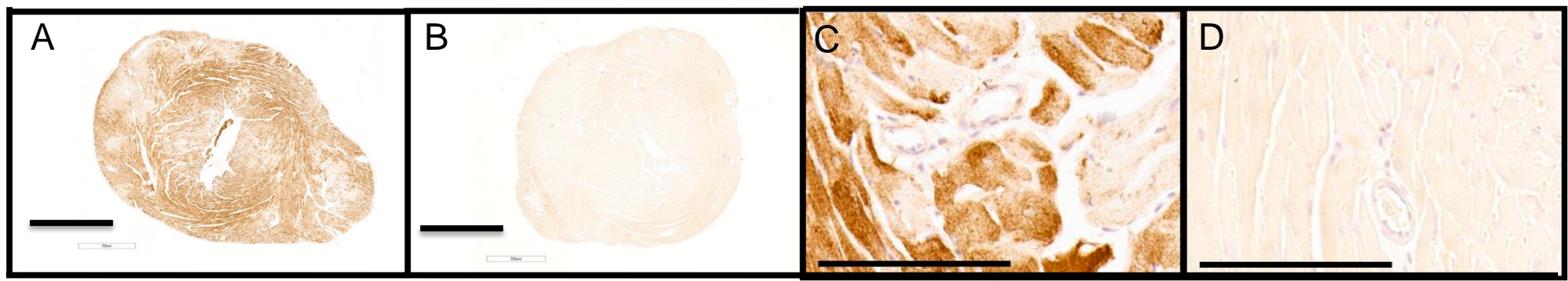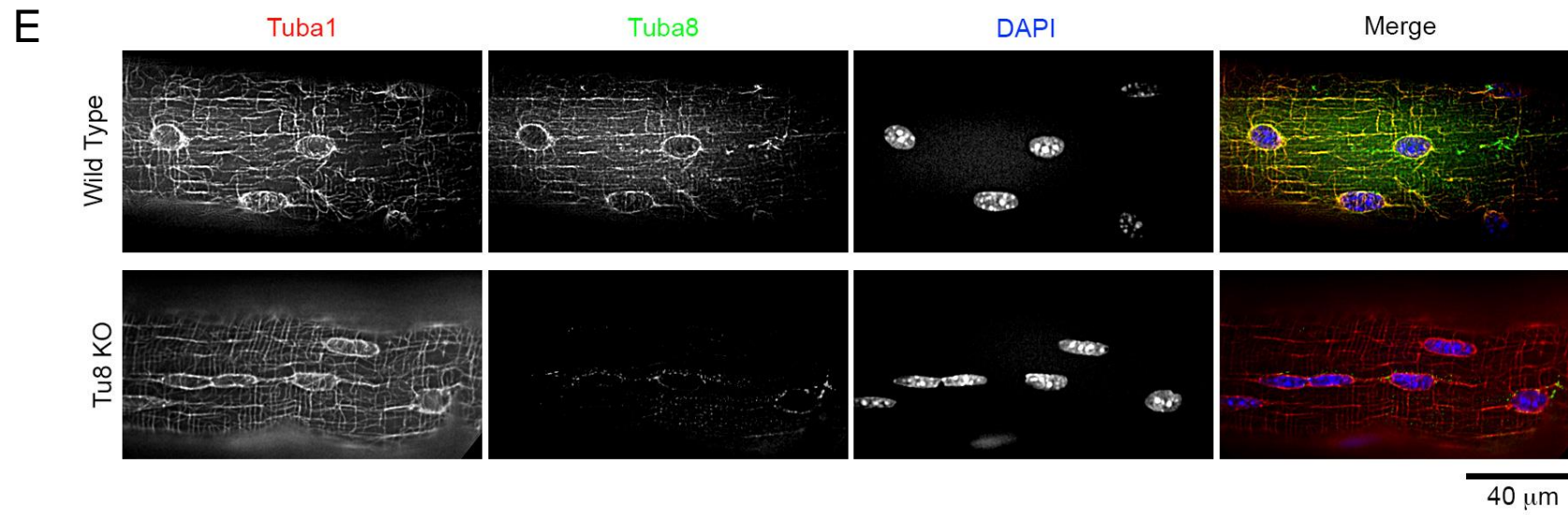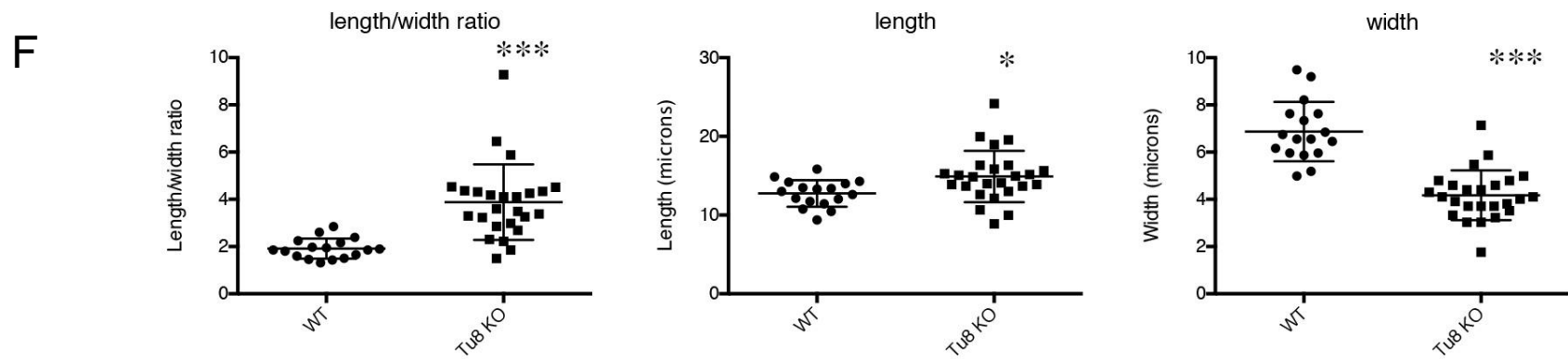

Supplement: S5 Fig — (PDF) [file pone.0174264.s005.pdf]

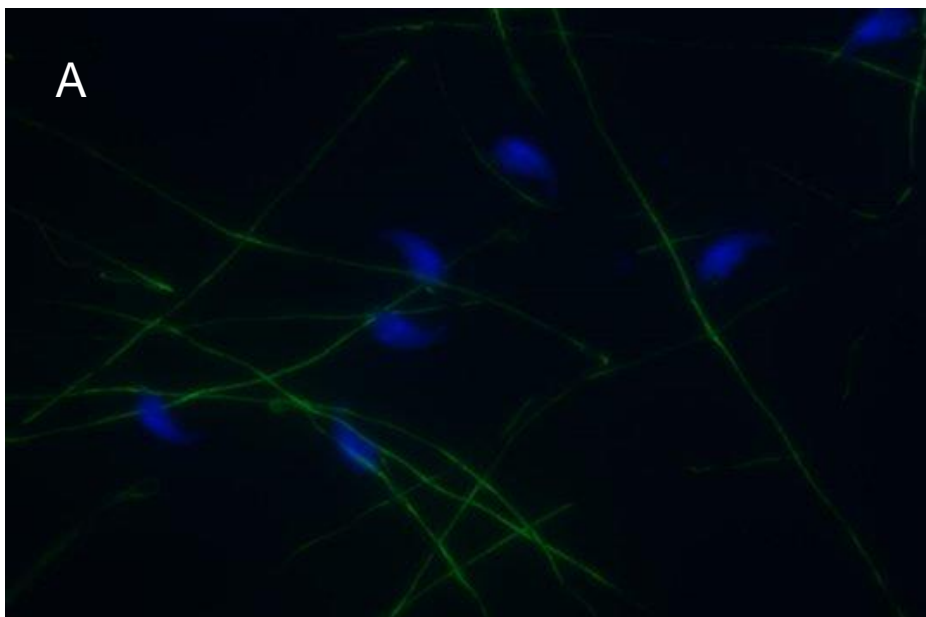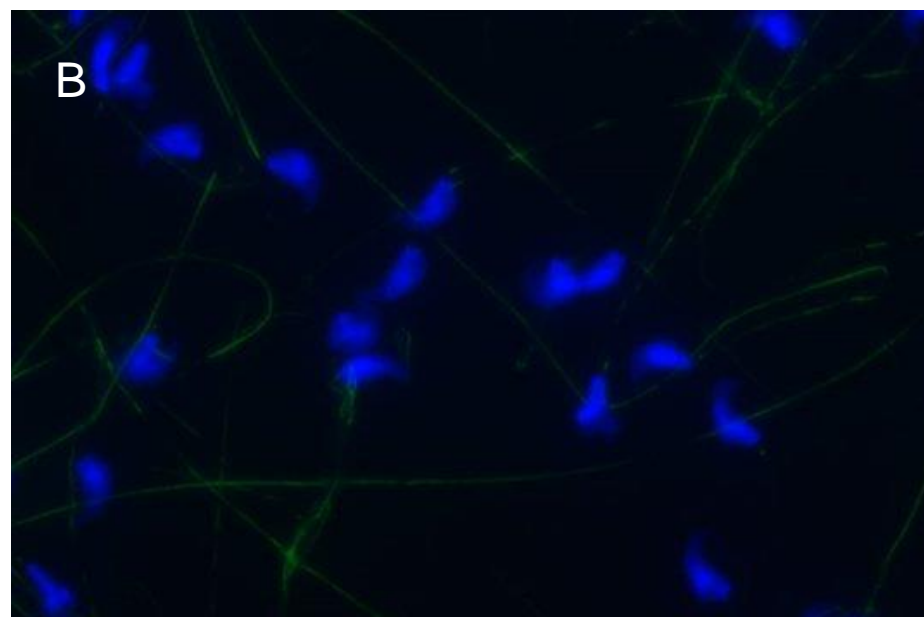

Supplement: S6 Fig — (PDF) [file pone.0174264.s006.pdf]

A

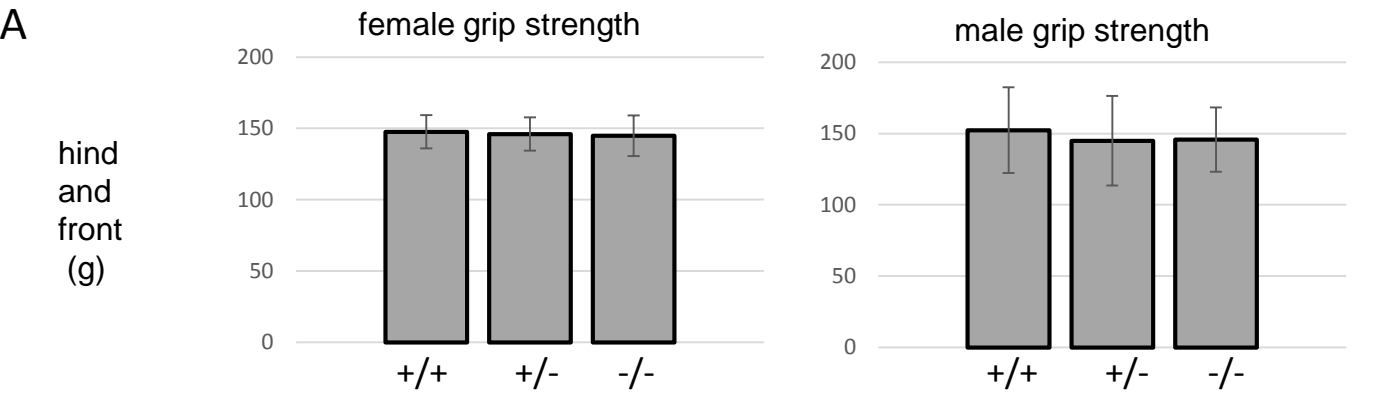

B

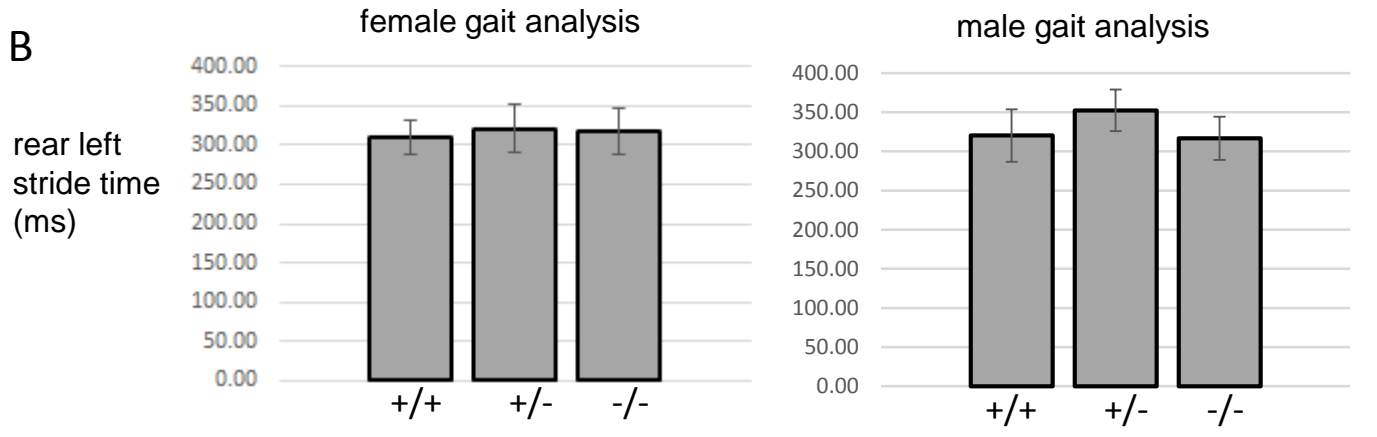

C

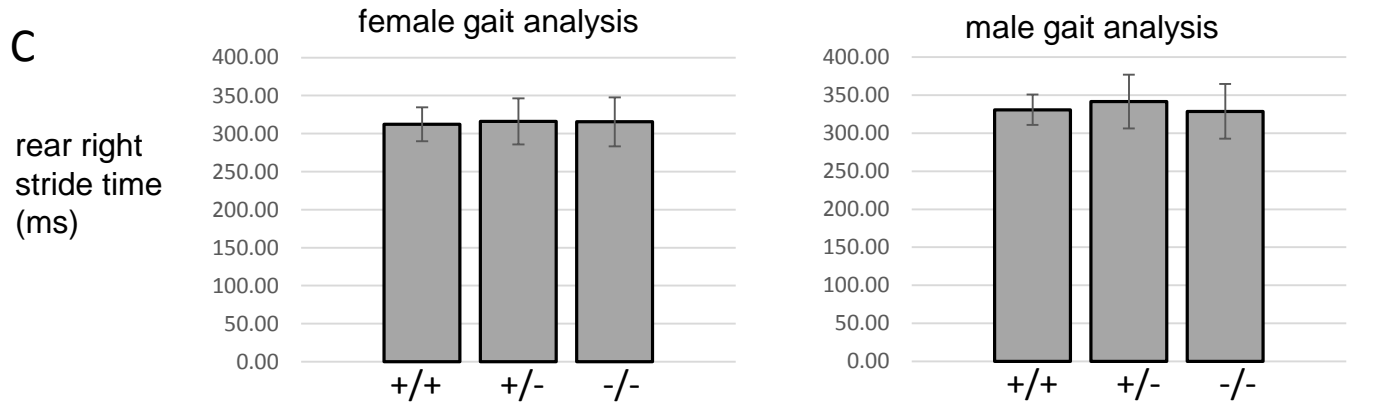

D

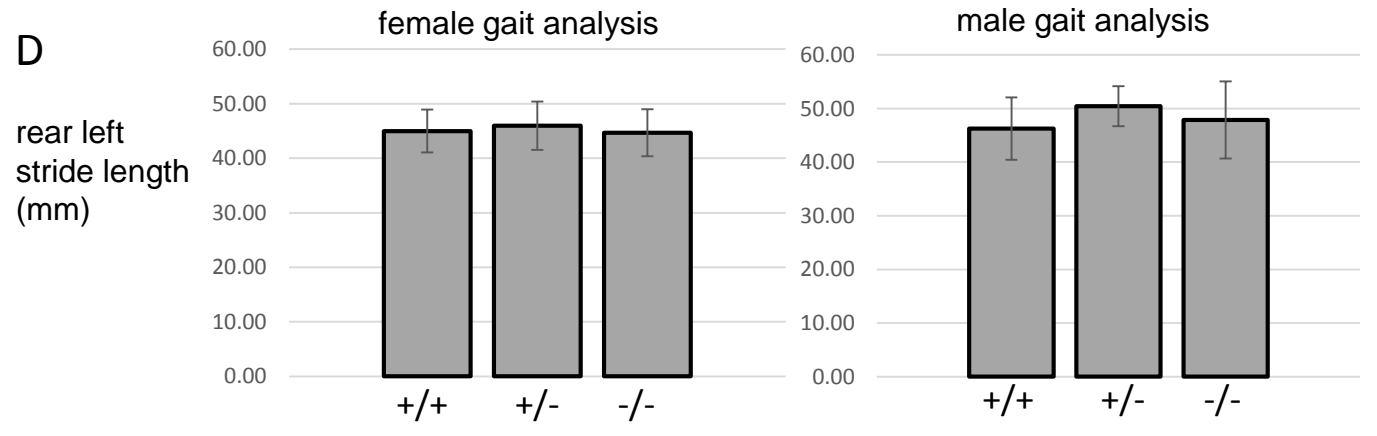

E

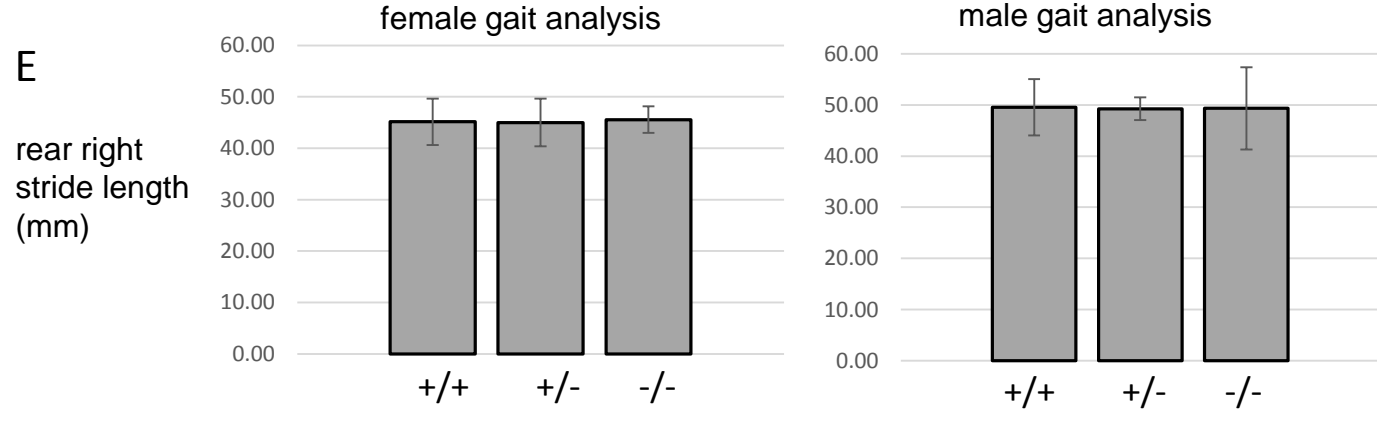

Supplement: S7 Fig — (PDF) [file pone.0174264.s007.pdf]

**A**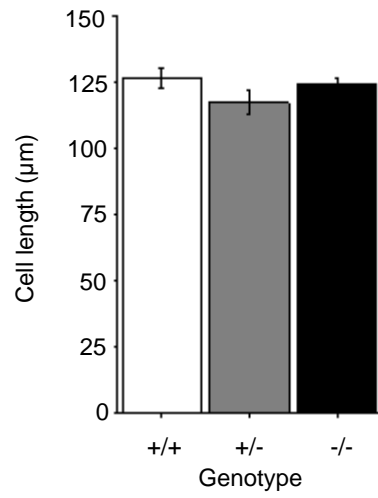**B**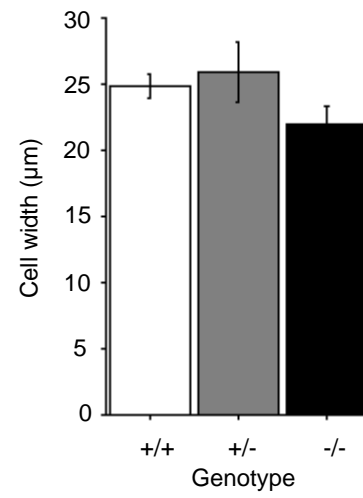**C**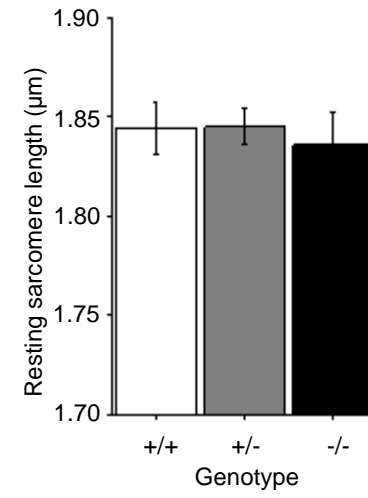**D**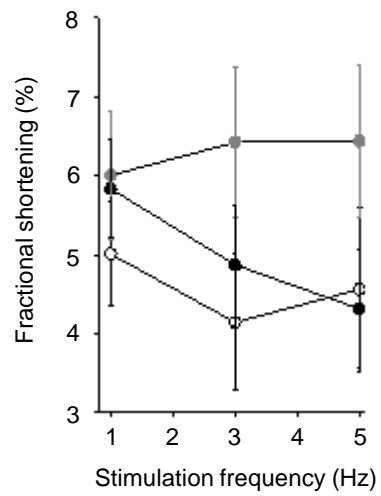**E**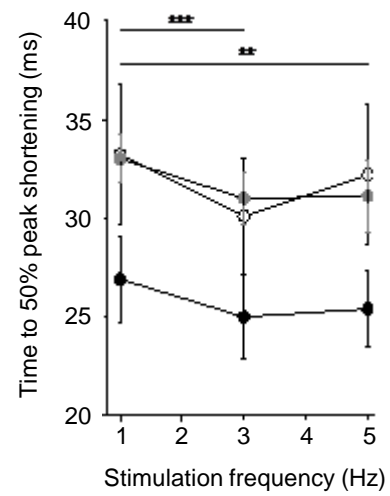**F**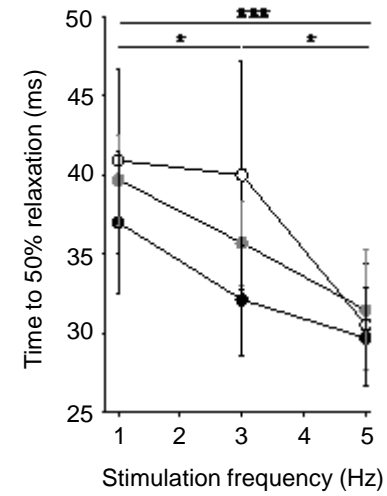

Supplement: S8 Fig — (PDF) [file pone.0174264.s008.pdf]
