# Supplementary material for: A tubulin alpha 8 mouse knockout model indicates a likely role in spermatogenesis but not in brain development
Source: PLoS One. 2017 Apr 7;12(4):e0174264. doi: 10.1371/journal.pone.0174264 (PMC5384676; doi:10.1371/journal.pone.0174264)
Supplement: S1 Table — (PDF) [file pone.0174264.s009.pdf]

S1 Table. Summary metrics for exome sequencing assay

Sample ID	Total raw read pairs	Read pairs passing quality filters (%)	Reads identified as duplicates (%)	Total mapped reads*	Exome mapped reads	Reads on target (%)
1	56,307,492	55,394,418 (98.4%)	9.67	100,037,913	57,361,609	57.34
2	59,470,023	58,532,142 (98.4%)	9.15	106,300,087	60,172,705	56.61

* Following duplicate removal
