# Supplementary material for: A tubulin alpha 8 mouse knockout model indicates a likely role in spermatogenesis but not in brain development
Source: PLoS One. 2017 Apr 7;12(4):e0174264. doi: 10.1371/journal.pone.0174264 (PMC5384676; doi:10.1371/journal.pone.0174264)
Supplement: S2 Table — (PDF) [file pone.0174264.s010.pdf]

S2 Table. Variant count for each patient following sequential filtering criteria

Filtering criteria	Sample 1	Sample 2
Total variant count	33,283	33,951
Exons and splice site variants (+/- 20bp)	30,223	30,875
Homozygous variants located in the autozygous region reported in Abdollahi et al 2009. (chr22:17841068-25248310)	80	82
Exclusion of variants with a dbSNP or NHLBI GO Exome Sequencing Project MAF ≥0.01	15	17
