# Supplementary material for: A tubulin alpha 8 mouse knockout model indicates a likely role in spermatogenesis but not in brain development
Source: PLoS One. 2017 Apr 7;12(4):e0174264. doi: 10.1371/journal.pone.0174264 (PMC5384676; doi:10.1371/journal.pone.0174264)
Supplement: S1 Appendix — (DOCX) [file pone.0174264.s013.docx]

**S1 Appendix. Supporting Information**

**Material and Methods**

**Primer sequences**

Primers for generating the 500-bp external probe by PCR for Southern blot analysis:

Tuba8-Sth1 dGCCCATCTGCTATCTGTGTTC

Tuba8-Sth2 dAAGGACCCATCTTTCAGAAGC

Primers for genotyping flpe-containing alleles at the Rosa locus used 3-primer PCR:

GT1 dAAAGTCGCTCTGAGTTGTTAT

GT2 dGCGAAGAGTTTGTCCTCAACC

GT3 dGGAGCGGGAGAAATGGATATG

Primers for detection of the Cre recombinase gene were those described by Diggle et al [1].

Primers for genotyping the Tuba8 locus:

P1 dGACCTCACAAACAGCCCATT

P2 dATCCCTCTCGTCTGAACCTG

P3 dGGTTAGGCATGGTCTCAGGT

P4 dAGGCCTGGGAGGTAAACTGT

Primers for RT-PCR:

Exon2F dATGGAACCTTTGGCACTCAG

Exon4R dCGCTCCATCAGCAGAGAAGT

Primers for quantitative RT-PCR:

Tuba8 primers were those described by Braun et al 2010.

Hprt forward primer: dCGTCGTGATTAGCGATGATGA

Hprt reverse primer: dTCCAAATCCTCGGCATAATGA

**Immunohistochemical analysis of cortex layers**

For histological analysis of the brain, tissue was fixed overnight in 4% paraformaldehyde in PBS, embedded in 4% low melting point agarose, and 50 μm slices were cut coronally with a vibrating microtome (VT1000S, Leica). Immunostaining was performed from three litter-matched brains of each genotype, and on at least 3 slices per brain. Sections were incubated for a minimum of 1h in blocking solution (4% BSA; 3% Goat’s serum; 0.1% T-X-100) at RT, followed by overnight incubation at 4˚C with primary antibody diluted in blocking. Ctip2 clone 25B6 (Abcam) and Cux1 CDP (M- 222) (Santa Cruz Biotechnology). After 3 washes with PBS, secondary antibody diluted in blocking solution was added and incubation was carried out for 1-2h at RT. After another 5 min wash with PBS, slices were stained for 15 min with DAPI, washed again with PBS and mounted with ProLong Gold antifade reagent. Images were taken on a Zeiss LSM710 confocal.

**Single muscle fibre analysis.**

Fibres were isolated from two wild type and two knockout animals, and the method was essentially that described previously [1-2]. Briefly, the gastrocnemius was dissected from the hindlimb and transferred to a solution of sterile-filtered 2mg/ml collagenase Type I from *Clostridium histolyticum* (Sigma) diluted in isolation media (DMEM supplemented with 1% penicillin-streptomycin (P/S) (10,000 U/ml (Gibco). Muscle was digested at 37°C for 1hr 20 minutes then flushed with media, using a wide bore glass pipette coated with 10% horse serum diluted in PBS, to release fibres. Single fibres were selected with a coated narrow-bore glass pipette and washed three times in isolation media.

Single fibres were fixed with warmed 4% paraformaldehyde for 20 mins then washed three times for 3 min with PBS. Permeabilisation in 0.5% Triton X-100 in PBS was for 10 minutes, before washing three times in PBS. Blocking was for 30m mins in 20% horse serum followed by 2 hours in PBS diluted primary monoclonal antibodies (TUBA8 (SAB5300189, Sigma), tyrosinated alpha tubulin (YL1/2, ADSerotec), then washed three times in PBS with 0.025% Tween (PBST). Secondary antibodies were prepared in PBS and incubated for 1hr 30min, washed three times for 3 minutes in PBST, with a final PBS wash. Fibres were carefully selected with a fire-polished, narrow-bore glass pipette coated with 10% horse-serum and placed on a clean microscope slide. Excess PBS was removed and fibres air dried slightly before mounting in ProLong Gold antifade.

Images were taken on a Delta vision deconvolution microscope, using an Olympus 40X/1.35, UApo/340, IX70, 1-UB768 objective. A Z-stack of 8 images was taken then deconvolved, with a single deconvolved image used for nuclei width and length measurements using Image J.

**Mouse phenotyping**

Behavioural phenotyping was performed on a cohort of ten adult animals of each genotype. The age of mice used in the tests was as follows: openfield (10 weeks), modified SHIRPA (11 weeks), grip strength (11 weeks), spontaneous alternation (12 weeks), gait analysis (13 weeks), acoustic startle (13 weeks), motor function wheel running (weeks 14-16), fear conditioning ( 17 weeks). The wheel running was undertaken in motor function cages, following which the males were singly rehoused. All tests were undertaken at the MRC Mary Lyon Centre (Harwell, UK), using their standard operating procedures (S.O.Ps), which can be obtained from: <http://www.har.mrc.ac.uk/services/phenotyping>. Phenotyping data is in S1 and S2 data. Analysis used a one way ANOVA with Tukey multiple testing correction.

**Cardiomyocyte functional analysis**

Ventricular myocytes were isolated from two mice of each genotype group essentially as previously described [4], modified by the inclusion of 10 µl/ml heparin (Sigma, 1,000 IU/ml) in the initial flushing solution, 10 mM 2,3-butanedione monoxime in the subsequent isolation solutions, and no added Ca^2+^ in the storage solution.

For experiments the isolated myocytes were superfused with a physiological saline, containing 137 mM NaCl, 5.4 mM KCl, 0.33 mM NaH_2_PO_4_, 1.8 mM CaCl_2_, 0.5 mM MgCl_2_, 5 mM HEPES, 5.6 mM glucose, adjusted to pH 7.4 with NaOH at 37 ˚C. Cells were stimulated to contract via external platinum electrodes. Stimulation frequency was 1, 3 and 5 Hz. A video image of the cell was used to measure resting cell dimensions and sarcomere length via IonWizard software (Ionoptix, Milton, MA). The amplitude and time course of myocyte contraction were also measured in this way. The index of contractility was shortening, expressed as a percentage of resting length. The morphology of the myocytes was tested by one-way ANOVA and the contractile parameters by two-way repeated measures ANOVA (the factors were Tuba8 group and stimulation frequency) followed by Tukey multiple comparison tests.

**References:**

1. Diggle CP, Shires M, McRae C, Crellin D, Fisher J, Carr IM, et al. Both isoforms of ketohexokinase are dispensable for normal growth and development. Physiol Genomics. 2010;42A(4):235-43.
2. Keire P, Shearer A, Shefer G, Yablonka-Reuveni Z. Isolation and culture of skeletal muscle myofibers as a means to analyze satellite cells. Methods Mol Biol. 2013;946:431-68.
3. Pasut A, Jones AE, Rudnicki MA. Isolation and culture of individual myofibers and their satellite cells from adult skeletal muscle. J Vis Exp. 2013;(73):e50074.
4. McCrossan ZA, Billeter R, White E. Transmural changes in size, contractile and electrical properties of SHR left ventricular myocytes during compensated hypertrophy. Cardiovasc Res 2004; 63: 283–292.

**Figure Legends**

**S1 Figure. Targeting of the Tubulin alpha 8 (Tuba8) gene.**

1. Southern blot design. Vertical arrows represent the extent of the construct between the SbfI and NgoAIV sites. By insertion of the neomycin cassette with FLP and LoxP sites into the PspoMI site, only one PspOM1 site was retained. The double-headed arrow represents the location of the external probe. PspOMI digestion was used to screen the ES cell DNA for correctly targeted construct, in combination with a radiolabelled probe external to the construct itself. The neomycin cassette was approximately 2 kb, and the wild type allele was 7.2 kb whilst the correctly targeted allele was 9.1 kb.
2. Southern blot analysis of ES cell clones. Twelve PspoMI-digested ES cell DNAs were separated on a 0.8% agarose gel along with a molecular weight marker lambda HindIII (lane 13). The external probe identified a wild-type DNA fragment of approximately 7.2 kb, whilst correct homologous recombination labelled a larger product of 9.1 kb. Nine ES clones had the correctly inserted vector.
3. The genotyping strategy used four primers to amplify differentially sized PCR products dependent on the genotype. The positions of the primers (P1 to P4) are indicated by the dashed arrows.
4. An example of a genotyping PCR results using primers P1 and P2. Samples 1, 3 and 4 are heterozygous for the floxed allele, sample 2 is homozygous and sample 5 is wild type.

**S2 Fig. Loss of Tuba8 RNA expression in knockout mice.**

1. Standard RT-PCR on skeletal muscle from 3 genotypes (+/+, +/-, -/-). PCR was performed on either (1) reverse transcribed, or (2) non-reverse transcribed, skeletal muscle RNA. Primers were situated in exon 2 and exon 4 generating a PCR product of 370 bps in the wild type transcript, whereas the exon 3 deleted transcript generated a smaller 221 bps product. The heterozygous sample contained both trasncripts.
2. Sanger sequencing of the wild type (+/+) and the deleted (-/-) transcript in skeletal muscle. The vertical arrow indicates the splice junction, and showed exon 2 and 3 correctly spliced in the wild type transcript. In the knockout transcript exon 2 was spliced directly to exon 4, that generated a new stop codon close to the start of exon 4.
3. Quantitative RT-PCR on skeletal muscle from the three genotypes (+/+, +/−, −/−). Four animals of each genotype were used. Tuba8 levels were normalised against Hprt levels, and plotted as the average of the four animals, with error bars representing ±1 s.d. Unpaired t-test showed statistically significant differences between the genotypes, p<0.05

**S3 Fig. Loss of Tuba8 protein expression in knockout mice**

A. Quantification of testis Tuba8 expression by western blotting. Membranes with separated whole testis extracts from each of the three genotypes were immunolabelled for Tuba8 expression (Bioserv), and re-probed for Gapdh expression. This was repeated four times using lysates from different animals. Tuba8 expression levels were normalised to Gapdh levels, and wild type levels set at 1.0 to assess relative expression between the genotypes. The graph shows average relative expression and error bars represent ±1 s.d. Tuba8 levels were statistically different, p<0.05 comparing heterozygous and knockout samples using an unpaired t-test.

B. An example of a western blot in which whole brain extracts were immunolabelled for Tuba8 (Bioserv). A specific band of the appropriate size of approximately 55 kDa was identified in the normal brain lysate.

C. Testis lysates were separated on an 18% SDS-PAGE gel prior to western blotting. Immunolabelling used (1) the Bioserv primary antibody, as the TFGTQASKIND epitope used for antibody generation was present in exon 2, therefore it should be able to detect the 76 amino acid truncated protein. A no primary antibody control was also used (2). No 8 kDa protein product was detected in the knockout sample.

**S4 Fig. Normal cortical lamination in Tuba8 deficient brains.**

(**A,B**) Immunostaining with anti-Ctip2 (red) and anti-Cux1 (green) antibodies in E18.5 embryonic brains of Tuba8 heterozygous (A) and homozygous (B) mutants. Cux1-positive upper layer neurons (A’, A’’’; B’, B’’’) are located on top of Ctip2-positive layer V neurons (A’, A’’; B’, B’’) indicating normal lamination. Some upper layer neurons are still migrating at this stage and appear as green cells throughout the cortical plate in both conditions (A’, A’’’; B’, B’’’). (**C,D**) Immunostaining with anti-Ctip2 (red) and anti-Cux1 (green) antibodies in P10 postnatal brains of Tuba8 heterozygous (C) and homozygous (D) mutants. Again, green labeled Cux1-positive neurons are located in layers II-IV (C’, C’’’; D’, D’’’) on top of red labeled Ctip2-positive neurons, which reside in layer V (C’, C’’; D’, D’’). Ctip2 labeled neurons in the CA1 and DG regions of the hippocampus also show normal distribution in both heterozygous and homozygous Tuba8 mutants. Cortical layers are labelled with roman numbers (II to VI); WM, white matter; DG, dentate gyrus; CA1-CA3, Cornu Ammonis areas 1-3. Scale bars = 100 μm, except (A’-A’’’ and B’-B’’’) = 50 μm.

**S5 Fig. Tuba8 localisation in muscle**

Immunohistochemistry was performed on adult mouse hearts using a monoclonal antibody against Tuba8 (Sigma). Sectioning was from the apex of the heart, and sections were taken when the lumen of the left ventricle was visible. **A.** Whole wild-type heart; scale bar = 1mm. **B.** whole knockout heart. **C.**.Part of a wild type heart at higher magnification; scale bar = 100µm **D.** Part of the knockout heart at higher magnification.

**E.** Immunocytochemistry on wild type and Tuba8 knockout (Tu8 KO) isolated striated muscle fibres, showing localisation of Tuba8 (merged image in green), tyrosinated alpha tubulin (Tuba1, merged image in red), with DAPI stained nuclei (merged image in blue). **F**. Analysis of nuclei size from 4 fibres for wild type and 7 fibres for Tu8 KO with 3-5 nuclei analysed per fibre. Error bars represent one standard deviation.

**S6 Fig. Tuba8 in mouse sperm**

Immunocytochemistry on control (A) and knockout (B) mouse sperm fixed with ice cold methanol using the anti-TUBA8 antibody from Sigma.

**S7 Fig. Behavioural phenotyping**

1. Grip strength analysis in female and male mice. No significant difference was identified.
2. Stride time of the rear left limb generated from the gait analysis for female and male mice. No significant differences were identified in the female mice. A significant difference was found in the male mice between the wild type and heterozygous animals, but not between the wild type and homozygous animals.
3. Stride time of the rear right limb generated from the gait analysis for female and male mice. No significant differences were identified in the female or male mice.
4. Stride length of the rear left limb generated from the gait analysis for female and male mice. No significant differences were identified in the female mice. A significant difference was found in the male mice between the wild type and heterozygous animals, but not between the wild type and homozygous animals.
5. Stride length of the rear right limb generated from the gait analysis for female and male mice. No significant differences were identified in the female or male mice.

**S8 Fig. Isolated cardiomyocyte analysis**

1. Myocyte length in wildtype (+/+), heterozygous (+/-) and homozygous knockout (-/-) myocytes. Twelve myocytes from two hearts were analysed from each genotype. Error bars represent SEM.
2. Myocyte width in wildtype (+/+), heterozygous (+/-) and homozygous knockout (-/-) myocytes. Twelve myocytes from two hearts were analysed from each genotype. Error bars represent SEM.
3. Resting sarcomere length in wildtype (+/+), heterozygous (+/-) and homozygous knockout (-/-) myocytes. Twelve myocytes from two hearts were analysed from each genotype. Error bars represent SEM.
4. Amplitude of contraction expressed as fractional shortening in wildtype (white circles), heterozygous (grey circles) and homozygous knockout (black circles) myocytes. Ten myocytes from two hearts from each genotype were used. Error bars represent SEM.
5. Time course of shortening expressed as time to half peak contraction. *** p < 0.001, ** p < 0.01, for pairwise comparisons. Wildtype (white circles), heterozygous (grey circles) and homozygous knockout (black circles). Error bars represent SEM.
6. Time course of relaxation expressed as time from peak to half relaxation. *** P < 0.001, * p < 0.05 for pairwise comparisons. Wildtype (white circles), heterozygous (grey circles) and homozygous knockout (black circles). Error bars represent SEM.
